# Development of Environmentally Clean Construction Materials Using Industrial Waste

**DOI:** 10.3390/ma15165726

**Published:** 2022-08-19

**Authors:** Galiya Zhanzakovna Alzhanova, Yelaman Kanatovich Aibuldinov, Zhanar Baktybaevna Iskakova, Saniya Manarbekkyzy Khabidolda, Gaziz Galymovich Abdiyussupov, Madi Toktasynuly Omirzak, Gunasekaran Murali, Nikolai Ivanovich Vatin

**Affiliations:** 1 Research Institute of New Chemical Technologies, L.N. Gumilyov Eurasian National University, 010008 Nur-Sultan, Kazakhstan; 2 Department of Chemistry and Chemical Technology, Kh. Dosmukhamedov Atyrau University, 060001 Atyrau, Kazakhstan; 3 Peter the Great St. Petersburg Polytechnic University, 195291 St. Petersburg, Russia

**Keywords:** blast furnace slag, red mud, lime production waste, natural loam stabilizing, structure formation processes, road base material, environmentally clean materials

## Abstract

The accumulated waste generated from industries severely affects environmental conditions. Using waste as a construction material or soil stabilization is an emerging area in the construction industry. Introducing new additive materials to strengthen local soils using industrial waste is an inexpensive and more effective method to improve the soil. In light of this, this study aims to develop environmentally clean construction materials for stabilizing natural loam (NL) using red mud (RM), blast furnace slag (BFS), and lime production waste (LPW). Nine different mixtures were prepared with four different combinations of RM (20, 30, and 40%), BFS (25, 30 and 35%), LPW (4, 6 and 8%), and various content of NL. X-ray diffraction (XRD), X-ray fluorescence (XRF), scanning electron microscopy (SEM), energy dispersive spectroscopy (EDS), atomic absorption spectroscopy (AAS), and axial compressive strength were examined. The results indicated that the optimum strength was obtained from the sample containing 40% of RM, 35% of BFS, and 8% of LPW. The observed compressive strength of the sample for 90 days was 7.38 MPa, water resistance was 7.12 MPa, and frost resistance was 7.35 MP, with low linear expansion meeting the demands for first class construction materials of the Kazakh norms. The mineral composition analysis evidenced the lack of heavy metals contaminants and hazardous compounds. Based on strength and environmental performance, RM, BFS, LPW, and NL mix can be used as a road base material. This process is believed to reduce environmental pollution related to RM and BFS, and lower the road base cost.

## 1. Introduction

There is a great interest in using industrial waste worldwide and a growing interest in using recycled materials in road construction. Using industrial waste, such as fly ash, metallurgical slags, plastic waste, and others, is common in many countries. The technical characteristics of such industrial by-products make it possible to replace some natural aggregates or binding materials in road construction [1,2]. Introducing industrial waste as building materials is one of the effective methods of using it in construction and eliminates waste disposal in large quantities [3]. Bauxite residue or red mud (RM) is an industrial waste generated in bauxite ore processing into alumina after strong alkali leaching. A study reported that the production of 1 ton of alumina produces 11.5 tons of RM [4]. About 120 million tons of red mud are produced worldwide each year, and it is estimated that 30 billion metric tons have already been accumulated worldwide [5]. Using waste materials to strengthen the soil is an emerging field in geotechnical engineering [6,7,8,9,10].

The RM mud is very caustic and its pH can be between 9.0 and 12.5. The high alkalinity storage of RM leads to serious environmental problems [11]. However, the alkaline characteristics of the bauxite residue reduce with enlarged duration following utilization: the cation exchangeability and the exchange capacity of sodium decrease with increasing post-disposal time [12]. RM may also contain elevated concentrations of natural radioactive elements [13,14]. RM can be used in various ways, such as building materials; adsorbents for heavy metal removal; and pigment production, and as a resource for recovering iron, titanium, and other trace metals [15]. Using significant amounts of red mud in road construction is one of the most common procedures. RM has limited practical applications in road construction, depending on the process conditions, refinery design, and bauxite quality [16]. However, comparative studies of the potential use of bauxite residues in road construction are still insufficient [17]. Using RM to produce building materials, including cement, ceramics, bricks, etc., can help solve environmental issues [5]. RM has low cost, high strength, and moisture resistance, and can be used as a road base material in China [18]. The use of bauxite residues to produce asphalt mixtures and road construction has attracted the attention of researchers. The potential use of coarse RM in the construction industry as road bases, embankments, and fillers for levees and seawalls. The tested sample contains 70% of coarse RM, 25% of fly ash, and 5% of lime. The results suggest that the stabilized residue outperformed the traditional material and can be used as a road base material with enhanced performance [19]. This is due to the strength of eco-friendly materials used for road bases with a content of 30% RM. Results indicated that the optimum proportions of sample strength after curing for 7 and 28 days were 5.92 MPa and 6.66 MPa, respectively. The observed strength is within the limits suggested by Chinese standards (5–7 MPa) [20]. The production of a road base material utilized raw components of RM, electrolytic manganese, and carbide slag. The findings revealed that the unconfined compressive strength after 7 days was 5.6 MPa. The excellent polymerized and pore structures were observed in road base materials under the synergistic action [21].

Ferrous slag is a by-product formed under high temperatures due to melting waste rock of iron-containing materials, flux, and impurities [22]. The chemical composition of ferrous slag mostly consists of oxides of iron, aluminium, silicon, magnesium, calcium, and manganese. The production process, raw materials, method, and cooling rate affect the number of substances formed [23]. Ferrous slags are classified into acidic and basic slags. Acidic slags contain more acidic oxides of silicon and phosphorus. Basic slags contain mostly basic oxides of ferrum, calcium, and magnesium. Some elements’ concentration in different slag types exceeds general environmental guidelines based on multiple exposure routes [24]. Studies have shown that the release of iron slag deposits has a detrimental effect on surface and groundwater [25]. Radioactive elements may be present in the composition of ferrous slag, for example, isotopes of potassium, radium, and thorium. Many studies have observed that large amounts of deposited slag can contaminate soil and groundwater by leaching toxic elements such as Cd, Cr, Cu, Pb, Ni, and Zn [26]. Ferrous slags have long been utilized as inert stones in the construction of road foundations. Ferrous slag is a very valuable resource and can be used in the construction of very high-quality road surfaces, as it has excellent technical and physical characteristics compared to a filler made of natural materials. Ferrous slag is disposed of in one tank by numerous factories. The accumulation of steel slag in landfills without treatment, which is caused by the release of heavy metals from steel slag, seriously pollutes rivers and agricultural land. There have been numerous attempts to safely reuse steel slag by lowering the danger involved.

Approximately 0.3–1 tons of blast furnace slag (BFS) is produced during the production of 1 tons of pig iron [27]. BFS and fly ash are used for road surface stabilization. It can also be mixed with lime to improve the strength and durability of some roads for superior pavement performance [28]. BFS has been used to replace coarse and fine aggregates in asphalt concrete mixtures [29]. Air-cooled slag is hard and has fine properties, so it is suitable as an aggregate. Adding granular slag to concrete increases its strength and durability since the slag has good cement properties. Granulated and expanded slag is practiced as a light filler in construction due to its low density [30,31]. BFS is also used to stabilize soft clay to increase its strength and can replace a high cement content in concrete production [32,33]. With this in mind, it is advisable to use recycled materials in construction to save space for landfill purposes, reduce carbon dioxide, and reduce waste-related costs [34,35]. Blast furnace slag has been established to have the ability to improve strength by interacting with lime to generate more C-S-H gel [36]. BFS is the major base material used in the alkali-activated binders [37].

Lime production waste (LPW) is generated when calcite or carbonate raw materials are burned at temperatures below 960 °C and can be sold as a by-product containing lime and other components (Al_2_O_3_, MgO, Fe_2_O_3_, and others). LPW is usually utilized to neutralize the soil’s acidity, make Portland cement, and disinfect wastewater. Due to its chemical interaction, LPW is employed as an alkaline activator of particle surfaces of different industrial and municipal wastes to generate novel composites and building materials [38]. Zhang and Li (2019) [39] investigated a stabilization of red mud comprising 22.5% of fly ash and 7.5% of lime. Findings indicated the excellent performance in temperature shrinkage, dry shrinkage, and freezing and thawing. The compression rebound modulus of developed materials is superior to commonly used materials. A comparative study of standard and admixture-based soil was carried out by Nitish Kumar (2022) [40]. The admixtures used were fly ash (10, 20, 30, and 40%), lime sludge (5, 10, 15, and 20%) and polypropylene fibres (0.25, 0.5, 0.75, and 1.0%). Results revealed that the addition of fly ash, which ranged from 20 to 30%, exhibited an excellent strength. The maximum shear strength was observed in soil with 20% fly ash and for California bearing ratio of soil with 20% fly ash. Fibers with 0.5 and 0.75% exhibited a good California bearing ratio and shear strength, respectively. In the case of lime addition, the maximum values found for California bearing ratio and shear strength were 20 and 10%, respectively.

Several literatures are available regarding the characterization of the individual materials. However, the different combinations of soil using these materials and their characterization were unexplored by any researchers. The pozzolanic reactivity of a component depends on the amorphous silica content, fineness, mixing ratio, available alkaline medium, and temperature [41]. To fill this research gap, this study aims to develop eco-friendly materials by replacing natural materials with blast furnace slag, red mud, and lime production waste. X-ray diffraction (XRD), X-ray fluorescence (XRF), scanning electron microscopy (SEM), energy dispersive spectroscopy (EDS), and axial compressive strength were examined. The main goals of this study are develop environmentally clean construction materials for stabilizing natural loam (NL) (composites with the highest possible fractions of industrial wastes RM, BFS) and LPW; and find new eco-friendly materials with mechanical qualities appropriate for civil engineering applications. Production wastes are used as a binder for NL for road base construction with complete replacement of traditional layers of natural sand and rubble. The current analysis shows that RM and BFS make up the majority of the combinations with LPW and NL (40 and 35%, respectively). Developed materials can be utilized for road base, municipal and industrial waste dumps, building foundations, tile and brick manufacturing, etc. These compositions’ novelty, high concentration of industrial waste, binding of heavy metals, and ease of manufacture make them superior to those found in the literature. With improved mechanical qualities, these composites can also be utilized to create bricks, blocks, and other types of construction materials. This study’s significance and urgency stem from the steadily rising level of environmental pollution caused by industrial wastes and the global increase in atmospheric temperature, which has recently resulted in environmental catastrophes across the globe.

## 2. Materials and Methods

### 2.1. Materials

**Figure 1:**
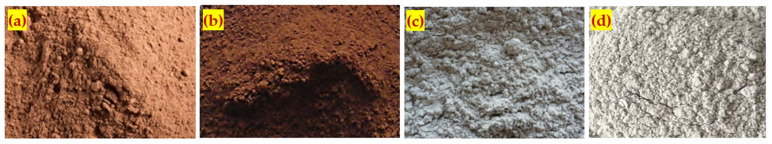
Sourced materials: (**a**) NL; (**b**) RM; (**c**) BFS; (**d**) LPW.

All materials were sourced from the different locations at Kazakhstan. The BFS is collected from ArcelorMittal Temirtau, Karaganda. The collected samples were crushed to lower the granulometry of the BFS. Red mud is collected from the Pavlodar aluminium plant, substandard lime from Lime Plant of Pavlodar region at Maikain. The loam soil sample is collected from a quarry to extract non-metallic materials near the city of Nur-Sultan. The materials were examined after a few days from the date of collection. The collected four materials are shown in Figure 1.

### 2.2. Sample Preparation and Mixing Combinations

**Figure 2:**
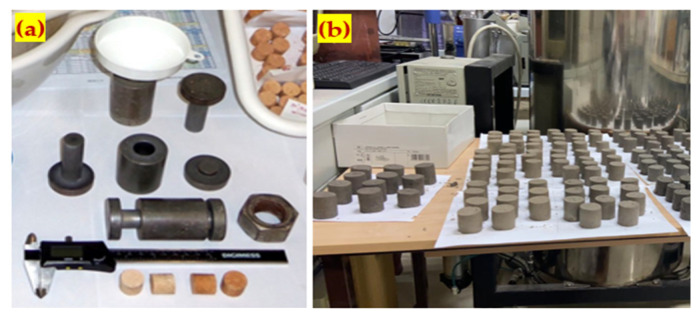
Details of specimen preparation: (**a**) tools used for compaction; (**b**) appearance of compacted specimens.

Every raw material sample was put through the drying process. The soft samples were pulverized in a porcelain mortar, and BFS was crushed in a ball mill and sieved through a 1.14 mm sieve. The dry mix of the materials was performed at various compositions, as demonstrated in Table 1. The mixing composition used in this study was selected based on the earlier research experience [42]. The mix id was labeled based on the content of RM, BFS, and LPW. For example, from the first mix (R20-B25-L4), R20 denoted the 20% of RM, B25 denoted a 25% of BFS, and L4 denoted a 4% of LPW. The other eight mix id were labeled in a similar way. In order to generate a 30 × 30 mm cylindrical sample, the mixtures were homogenized, hydrated at the optimal humidity (10–12%), compacted with a 10 MPa force, and hardened in outdoor circumstances. Figure 2a,b shows the tools used to prepare the specimens and the specimen’s appearance before testing, respectively. Nine different samples were arranged for pondering on 3, 7, 14, 28, 60, 90, 180, and 365 days of the test examples to calculate the midpoints, which add up to an amount of close to 900 samples. The samples were prepared from components taken in mass proportions, mixing RM, BFS, LPW, and NL at different ratios.

### 2.3. Methods

The following complementary techniques and research approaches were used to characterize the raw materials and final products to achieve the goal [43]. The mineral composition was studied using the X-ray Diffraction (XRD) from Philips PW 1830/40 Powder diffractometer (Caerphilly, United Kingdom). The machine was operated with an acceleration voltage of 40 kV and an X-ray beam current of 30 mA. Morphologically, the chemical composition of the raw materials was determined using an X-ray Fluorescence (XRF) Spectrometer Cu-tube (Philips Panalytical, Calgary, Canada) working with a characteristic *CuK_α_* wavelength of 1.5406 nm and machine is equipped with a Ni-filter. The elemental analysis of solubility and lixiviation of metals from liquid extracts was carried out by atomic absorption spectroscopy (AAS) on a Perkin Elmer 4100 spectrometer (Waltham, MA, USA). The SEM was performed using Ultra Plus (Carl Zeiss AG) to investigate the surface morphology of samples. The physical properties of the developed samples were examined for uniaxial compression, water, and frost resistance. Studies of changes in the strength of samples during the hydration of materials were carried out by studying the limit under uniaxial compression. The control of the change in the coefficient of linear expansion of materials during the hydration of samples was determined with a digital caliper. The calorimeter identified the chemical properties and carbonate content by changing the pH value. The electronic balance (ESJ201A, Zhejiang, China), ball mill (XQM-1. Hunan, China), and pH meter (PHS-25. Zhejiang, China) were used during the process.

The water-resistance coefficient was determined from the axial resistance strength of the samples on the 28th and 90th day. The change in the water resistance of materials after hydration and hardening after 28 and 90 days was determined in accordance with the requirements of the state standard of the Republic of Kazakhstan ST RK 973-94 «Stone materials and soils treated with inorganic binders for road and airfield construction».

The formula for calculating the water-resistance coefficient (*W_R_*) is given in Equation (1):

(1) $$W_{R}=\frac{R_{SAT}}{R_{AMB}}$$

*R_SAT_*—the uniaxial compressive resistance on the 28th day after sample total immersion in water for 24 h; *R_AMB_*—resistance under ambient conditions.

The water absorption values were calculated using Equation (2):

(2) $$W_{A}=\left[\frac{M_{SAT}-M_{D}}{M_{D}}\right]\times 100$$

*W_A_*—water absorption (%); *M_SAT_*—sample mass after 24-h water-saturation (g); *M_D_*—dry sample mass (g).

The formula for calculating the frost resistance coefficient (*C_FR_*) is given in Equation (3):

(3) $$C_{FR}=\frac{R_{F}}{R_{SAT}}$$

*R_F_*—the axial compressive strength after 25 freezing/thawing cycles, where each freeze occurs at negative 25 °C for 8 h; thawing also takes 8 h in water at positive 20 °C.

*R_F_*—the compressive strength after 24 h of water saturation at 20–25 °C.

The test measurements were measured employing a calliper, and the linear expansion coefficient (α) was calculated using Equation (4):

(4) $$\alpha =\left[\frac{D_{F}-D_{I}}{D_{I}}\right]\times 100\%$$

*D_I_*—initial diameter (mm); *D_F_*—final diameter (mm) [36].

## 3. Results and Discussion

### 3.1. Raw Materials Characterization

#### 3.1.1. Chemical Composition

Table 2 shows the major elements of resources by the XRF method. They mainly consist of Al, Si, Ca, and Fe. However, RM, BFS, and LPW contain more Ca than NL, whereas NL contains more Si than the rest. Both RM and BFS showed approximately the same total Ti, Al, and Mn content. The RM contains a large amount of iron (17.47%). Some iron compounds may contribute to the removal of copper [44]. The content of copper and other heavy metals in all components was not detected; the presence of manganese is negligible (by the AAS method).

The XRF examination of RM showed that most components are: CaO (45.15%), SiO_2_ (17.59%), Fe-oxides (25.39%), Al_2_O_3_ (4.57%), TiO_2_ (2.90%), and Na_2_O (2.81). These components are in good agreement with the other study [45]. According to studies, various toxic metals in RM concentrations range from 0.01 to 1% of the total weight [46], [47]. By AAS method (Table 3), the presence of heavy metals was found, such as Pb (0.17%), Zn (0.10%), Cu (0.05%), As (0.47%), etc. Heavy metals are well known for their carcinogenic and toxic properties. However, their content in RM is insignificant. Studies have reported that the presence of zinc in sediments has a more negative effect on the development of soil strength than other metals [48]. BFS is rich in CaO (37.75%) and pozzolans like SiO_2_ (31.86%) and Al_2_O_3_ (10.02%), and contains SO_3_ (2.67%). This is consistent with the findings of other studies by BFS [49,50]. Pozzolanic active phase (s) in any waste allows it to be used in practical applications [51].

The study of the values of leaching and solubility of metals from slag showed no excess of the maximum permissible concentrations for any chemical element (Table 4). NL mostly consists of SiO_2_ (56.45%) and concentration of Al_2_O_3_ (16.41%), CaO (11.43%), and Fe_2_O_3_ (7.94%). A noticeable content of FeO + Fe_2_O_3_ gives the soil a light brown color. The chemical composition of the LPW used as an activator content is 98.52%, and the impurities content of 1.51%. The raw materials contain more active mineral components, such as 2CaO SiO_2_, commonly used in construction materials. The main component of LPW is calcium hydroxide, which supplements sufficient calcium and hydroxide. In an alkaline environment, the compounds react with calcium hydroxide and as a result, cementing materials are formed, which determine the increase in the strengthening of the road foundation. The detailed chemical composition of the raw materials is shown in Table 3.

The pH values of RM, BFS, LPW, and NL are shown in Table 5, indicating that the alkalinity of RM is stronger than that of BFS, which may affect the hydration reaction. Alkaline minerals, such as calcite, tricalcium aluminate, apatite, hydrocalumite, and other minor components, contribute to RM’s alkalinity [52]. One of the most important components in soil system stabilization is pH. If the pH of the soil is more than 7, it has good reactivity with lime. In our case, natural loam is closer to neutral. The pH of the soil may differ based on the mine’s properties [53]. It is effective for the dissolution of silica and alumina at a higher pH, resulting in an increase in pozzolanic reaction, which contributes to greater flocculation. The alkali elements will be incorporated into the CSH phases over time and with increasing hydration, at least in the phase stabilization field of CSH with a pH greater than 9.5 [54]. Furthermore, as the pH rises, the hydrolysis of Si-O accelerates. Slag dissolution releases more aluminum ions, promoting the formation of ettringite.

#### 3.1.2. Structure of the Components

The analysis of the granulometric composition (Table 6) showed that more than half of the RM particles have a size of 1–2 microns and 10–50 microns. RM particles are characterized by high specific surface areas ranging from 15 to 58 m^2^/g [55]. RM has a low settling rate, low hydraulic conductivity, and a relatively high water-holding capacity because of its large specific surface area [49]. The number of particles approaching 1 mm in size (250–1000 microns) is only 5%, and exceeding the size of 1 mm is 2%.

#### 3.1.3. The Morphological Structure of the Components

**Figure 3:**
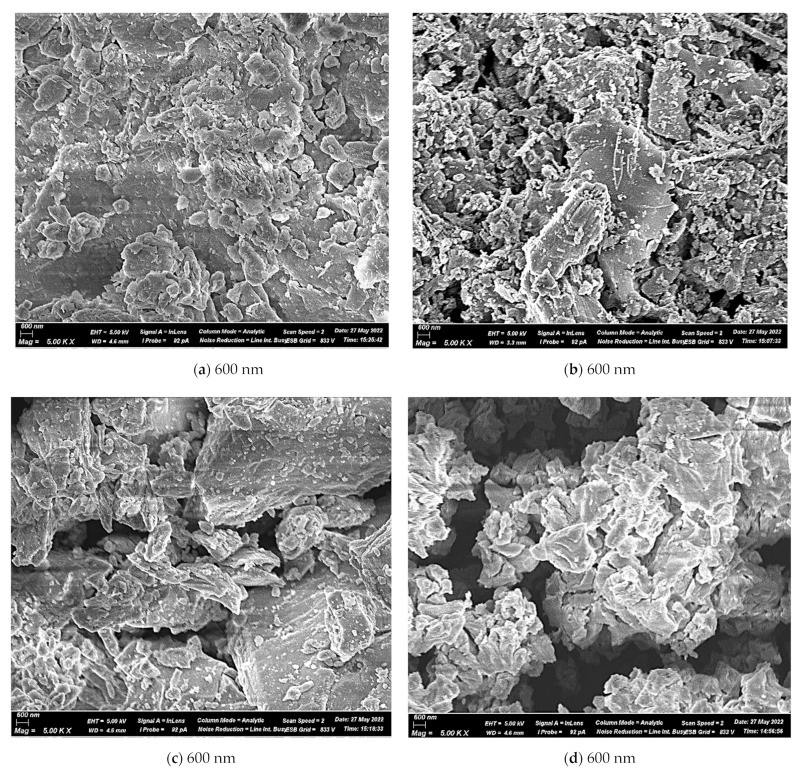
The SEM images of the (**a**) NL, (**b**) RM, (**c**) BFS, (**d**) LPW.

Figure 3 shows the microscopic images of raw materials obtained by SEM. The composition of the RM used includes a large number of iron oxides (27.39%), which explains the brown color of the bauxite residue used. The other components did not differ in color and appearance. All raw materials have a microstructure of shapeless microparticles of various sizes. The differences in materials in terms of morphology and particle size can be seen. It can be seen in the figure that raw materials are powdery substances that are agglomerated. RM has small irregular particles. Most of the samples’ surfaces are depicted as flow-rounded aggregates of varied sizes and shapes that are chemically unrelated; the pores between them are also of diverse sizes and shapes. According to micrographs, such a structure has amorphous compounds. The crystalline components identified by XRD may be hidden under the upper amorphous layer or have thin dimensions that cannot be distinguished with such magnification. BFS particles came in various sizes and forms, with some having sharp edges and morphology modification was evident by milling. As shown in Figure 3b, the degree of disorder on slag particle surfaces increased with scratches and uneven surfaces. The LPW particles have round forms with various diameters, as is typical of amorphous materials. NL has a homogeneous surface covered with cracks and rounded particles of various sizes.

**Figure 4:**
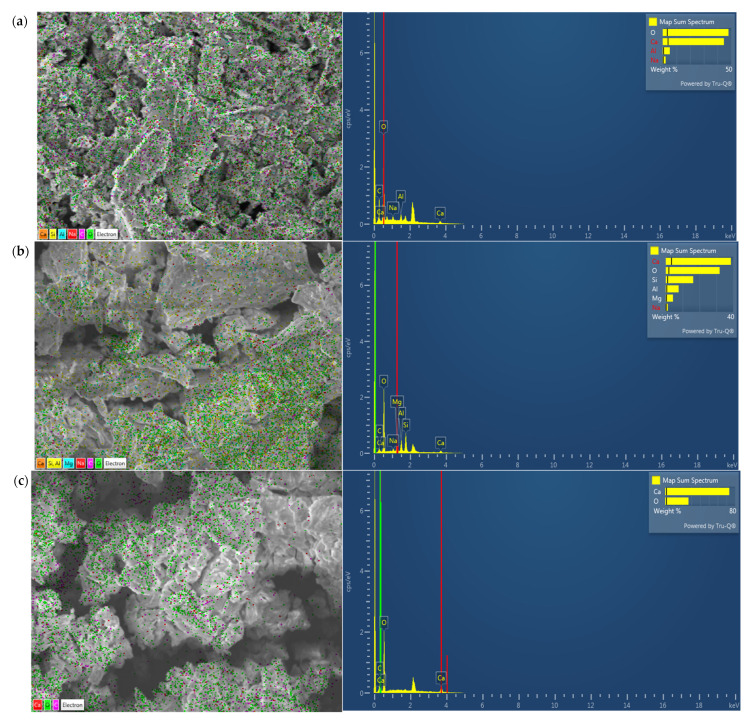

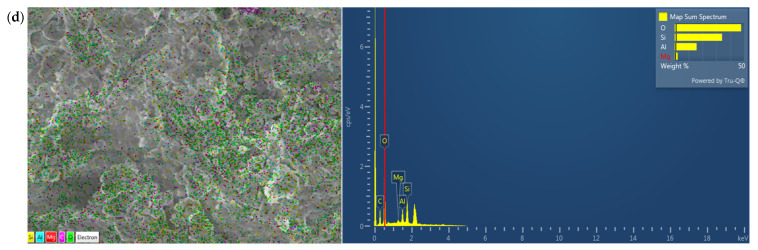
The SEM-EDS analysis of (**a**) RM, (**b**) BFS, (**c**) LPW, (**d**) NL.

The elemental composition of the spot on the sample surface of the raw material in Figure 4 was characterized by energy dispersive spectrometry (EDS). Micrographs with element distribution mapping on the sample’s focused surface allow researchers to examine high-relief surfaces, receive information about the state of the surface layer, and change the picture of sub-layers. The EDS graphs of the areas of RM points and the points of microchemical analysis by the EDS method showed the high content of chemical elements O, Ca, Al, Na, and C, respectively. This corresponds to Na_2_CaSiO_4_ and CaCO_3_ being surrounded by NaAlO_2_, which semi-quantitatively corresponds to CaTiO_3_ and SiO_2_. According to the EDS spectra, the main elements of BFS are Ca, Si, Al, Mg, Na, C, and O. This corresponds to the CaO-MgO-Al_2_O_3_-SiO_2_ system. The findings support the presence of the principal components determined by XRF: CaO, SiO_2_, MgO, Al_2_O_3_, and Na_2_O, together with minor admixtures of additional elements. The study of the micro-chemical composition of LPW showed a pronounced homogeneity of the chemical composition of the material at the nearest points. The primary components of LPW, represented by the elements Ca, O, and C (corresponding to CaO and CaCO_3_), are shown in Figure 4c. The presence of a large amount of amorphous phase NL can be determined by checking the chemical composition of crystal-like soil particles by the EDS method. The main elements included in the composition of NL are Si, O, Al, Mg, and C, corresponding to the content of quartz, calcite, and other minerals in NL. The SEM-EDS analysis results support these conclusions.

#### 3.1.4. Mineral Composition

**Figure 5:**
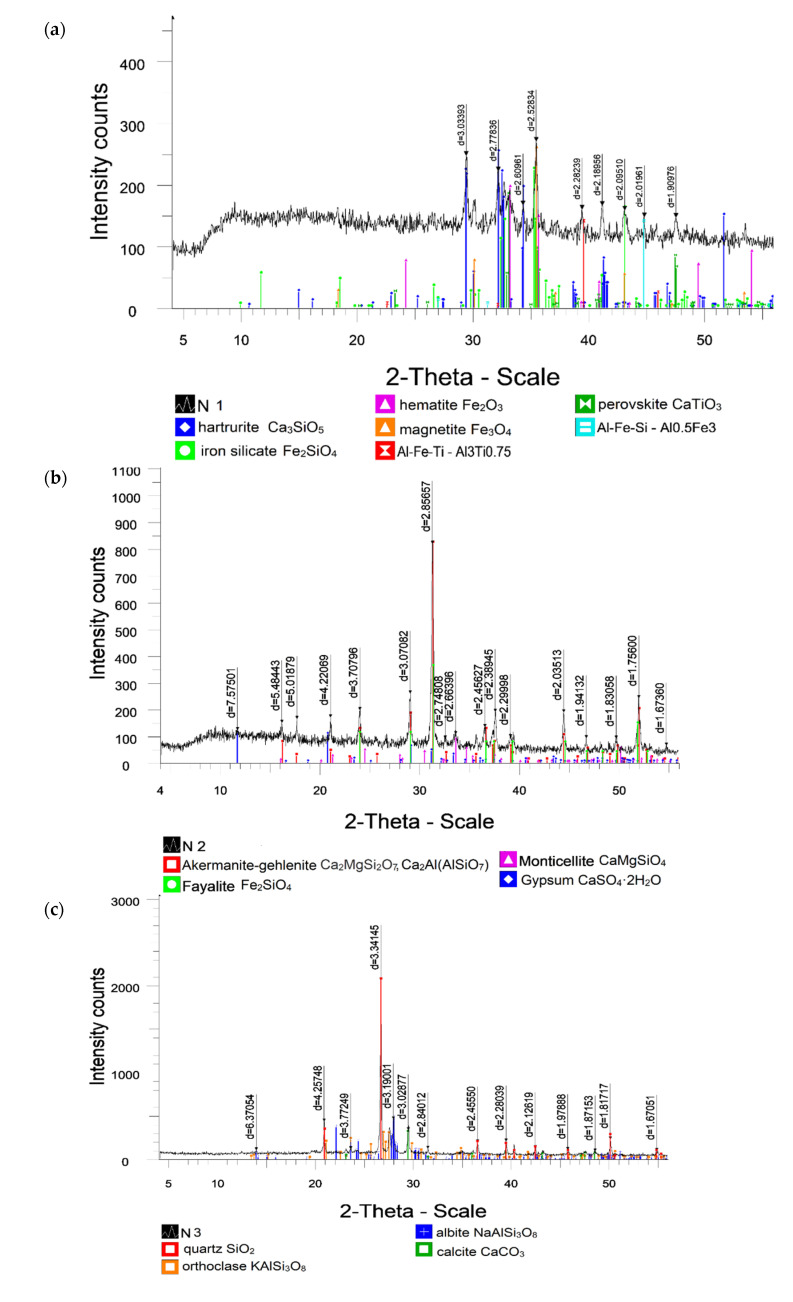

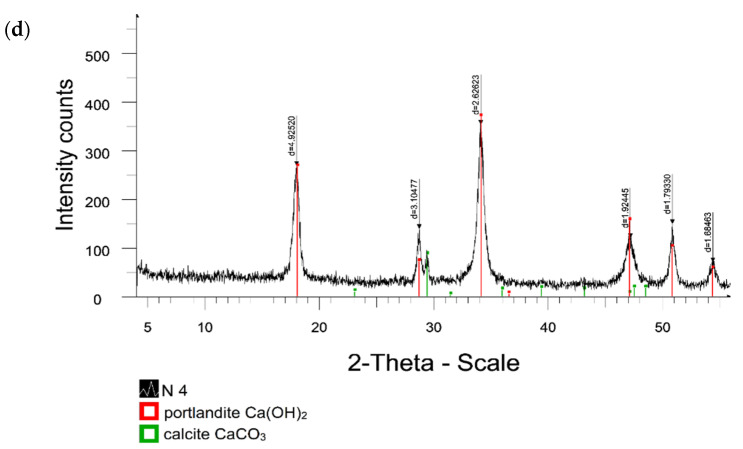
XRD analyses of (**a**) RM, (**b**) BFS, (**c**) NL, and (**d**) LPW.

On a semi-quantitative basis, X-ray phase analysis was performed on powder using a diffractogram of powder samples using the method of equal attachments and artificial mixtures. A quantitative ratio of crystal phases was determined. Characteristic diffraction reflexes allowing identification of the phases present are noted. In the research by Long et al. (2019) [56] and Gonzalez et al. (2021) [57], the mineralogical composition and microstructures of tests were decided by XRD and SEM. Figure 5 shows the XRD patterns for raw materials. The interpretation of diffractograms was carried out using data from the ICDD card file: the PDF2 powder diffraction data base (Powder Diffraction File) and diffractograms of minerals free of impurities. The diffraction peaks of RM were complicated and overlapped seriously comparing with the other crystal, and almost all phases with strong diffraction peaks were silicate mineral and iron-related phases. The major minerals detailed by researchers in RM are boehmite, kaolinite, quartz, rutile, diaspore, hematite, calcite, goethite, muscovite, and tricalcium aluminate [58,59,60]. Figure 3a shows the main mineral components of RM. The results of semi-quantitative X-ray phase analysis of crystalline phases show the presence of minerals like hatrurite (41.0%), iron silicate (22%), hematite (9.5%), magnetite (7.8%), perovskite (6.9%), and intermetallic compounds Al-Fe-Si (12.7%). The highest point of the peak shape appears at 2θ = 35.5°, and the diffraction peak was enriched with the higher peak-shape and the wider peak-surface between the angle of 29.4–35.9°, which further indicated that the content of silicate mineral in RM was relatively rich.

According to the XRD analysis, BFS represents a mixture of oxides of relatively complex chemical composition (Figure 5b); its composition was mainly presented by such mineral phases as calcite akermanite-gehlenite (66.3%), fayalite (23.7%), monticellite (5.6%), and gypsum (4.3%), typical for metallurgical slags. All XRD peaks of, except for the akermanite-gehlenite peak at 2θ = 31.2°, have a low intensity, indicating a low content of these minerals in the BFS. The second intense peak system at 2θ = 30° is related to fayalite. The amorphous phase represents the bulk of the sample, as evidenced by the large XRD background.

The clay minerals from NL (Figure 5c), include quartz (69.3, the highest peak shape appears at 2θ = 29.7°), albite (13.4%), calcite (10.2%), and orthoclase (7%). High amorphous phase content in the loam under study is characterized by a relatively low intensity scale and a very high X-ray background.

Figure 3d depicts the mineralogical phases of LPW. It can be seen that the main minerals are portlandite (85.8%) and calcite (14.2%). The most intense diffraction peak for portlandite is observed at diffraction angles, 2θ also equaling to 34.1°, I = 100%. This fact also suggests that the raw carbonate was stored under high humidity conditions after it was calcined. The second intense peak system is at 2θ = 29.5° and is related to calcite CaCO_3_, implying that the calcination temperature is insufficient for lime production as a raw material. Ca (OH)_2_, the major component of LPW, can supply enough calcium and hydroxide ions to generate an alkaline environment that promotes hydration. It is possible that the content of aluminum, calcium, and silicon oxides in the system can be activated by a hydroxide ion, which partially combines with a sulfate ion to form a burning substance that gives the system hydraulically cementing properties.

### 3.2. Physical and Mechanical Properties of the Developed Materials

This section presents the results of a study of changes in the physical and mechanical properties of samples as a result of their hydration and curing for 90 days. The values of the mechanical properties of the samples were calculated as the average and standard deviation of nine sample measurements.

#### 3.2.1. Axial Resistance of Test Specimens

**Figure 6:**
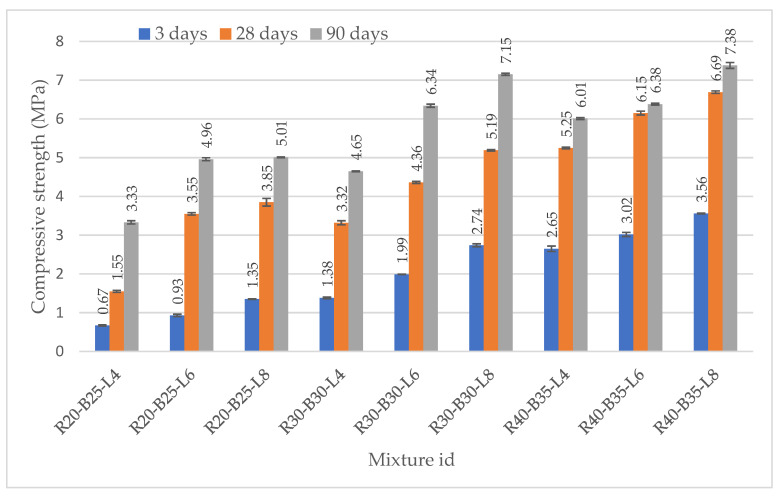
Changes in axial resistance of samples after 3, 28, and 90 days.

With increased concentrations of BFS, RM, and LPW, the strengthened materials’ different values grew gradually over time (Figure 6). The strength values of the samples were 0.67–3.56 MPa in 3 days, after 28 days 1.55–6.69 MPa, and 3.33–7.38 MPa after 90 days. The variations are barely noticeable and fall within the strength measures’ standard deviation. The collected experimental data’s axial resistance strength standard deviation values were never greater than 5% of the average means. The standard deviations of the test samples were low and varied between 0.005 and 0.10. The value of their strength is directly dependent on the content of each of the components. Composition R20-B25-L4 does not meet the requirements for practical use as a building material, since it demonstrated the least strength. The introduction of a 4% LPW additive as an activator of the astringent properties of slag does not give a fundamental increase in the strength of materials. Some materials with 6–8% LPW reached the strength of the first class already on the 28th day. The samples of composition R40-B35-L8 showed the best results among all the samples.

Figure 6 shows the compressive strength changes of test samples from 3 to 90 days. The addition of BFS, RM, and LPW improved compressive strength. Generally, when soil is mixed with RM, BFS, and LPW, there are chemical reactions that take place between them, which provide an improvement in strength. In any case, a few variables control the chemical responses, which give the change in quality-strength, such as moisture content, type of minerals, curing condition, weathering condition, and time. By the age of 90 days, the Kazakhstan building regulations for the strength of the first class (4–6 MPa) of first composition R20-B25-L4 with the disposal of three types of industrial waste meet for all compositions except one with minimum amounts of slag BFS (25%), RM (20%), and LPW (4%) with maximum soil content (51%). An increase in the content of LPW, RM, and BFS in materials invariably leads to an increase in their strength over time. This increase in strength leads to a decrease in the consumption of alkaline Ca and Mg ions to create and maintain high alkalinity necessary for the processes of alkaline excitation of surfaces of chemically neutral soil particles and slag particles with the removal of new amounts of Ca and Mg ions into the interstitial space. The strength of the best sample R40-B35-L8 was between 3.56 and 7.36 MPa after 90 days. Samples of the composition R40-B35-L8 already on the 3rd day have a strength of 3.56 MPa, which corresponds to the strength of the II-th class of road bases, and after 28 days these materials already correspond to the I-th class of strength of road bases (>6 MPa). In the previous investigation, NL was strengthened by LPW, ground-cooled ferrous slag [42]. The values also increased as the content of components and LPW increased over 90 days. The development of the C-S-H gel was aided by reactive CaO, SiO_2_, and Al_2_O_3_ in raw materials. Components react exceedingly slowly with water, resulting in the development of a hardening binder. According to the results and earlier studies, the compressive strength of stabilized soil samples increases with an increase in percentage of lime and of slag, and the compressive strength of stabilized soil samples increases with an increase in days of curing [36,61]. The compressive strength was increased using lime. Longer curing durations make the benefit more pronounced [62].

#### 3.2.2. Coefficient of Linear Dilatation and Shrinkage of Samples

**Figure 7:**
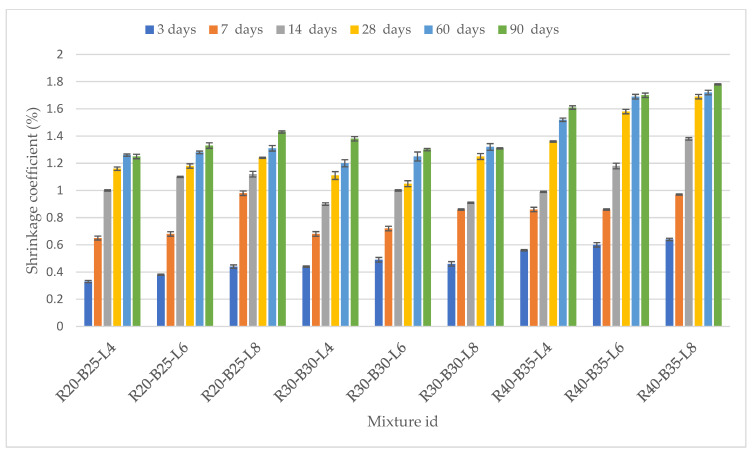
Changing the coefficient of linear expansion of composite samples.

The linear expansion coefficient of the samples was carried out on 3, 28, and 90 days, depending on the content of the components and the hydration time. Composites’ linear shrinkage is inversely proportional to their porosity. In other words, their coefficient of linear shrinkage decreases as their porosity increases. Values of the composites increased gradually (Figure 7) during the first 28 days, and the decreasing trend was observed until the 90th day, when values were between 1.15 and 1.72%. This is a more intensive shrinkage process compared to the previous age of the samples with the introduction of a large amount of LPW. Comparison of the difference in the expansion values at 3- and 90-day ages of each of the compositions shows that materials with a lower LPW content expand noticeably more than samples with a large amount of LPW. Perhaps this phenomenon is explained by a decrease in the stickiness of loam particles with a decrease in their number by 20–40%. Some samples’ shrinkage, which started 60 days before and continued to develop strength, shows compaction by tightening the material particles to the center. Perhaps this difference is explained by the difference in the slag’s chemical and mineral compositions. When the strength and expansion of samples are compared, it is clear that the maximal intensity of these processes of modifying the mechanical properties of samples occurs on the first day of hydration of mixes. If these processes had proceeded with the same intensity for all 90 days, the strength of the samples would have reached higher values. Samples with a lower NL content expand noticeably more than samples with a large amount of soil. Perhaps this phenomenon is explained by the decrease in the stickiness of NL particles with a decrease in their content (samples R40-B35-L4, R40-B35-L6, and R40-B35-L8). According to previous studies, the coefficient of linear expansion of samples begins to decrease due to gel compaction during syneresis. The standard deviations of the linear expansion coefficient of the test samples were low and varied between 0.003 and 0.016.

#### 3.2.3. Water and Frost Resistance of the Materials

**Figure 8:**
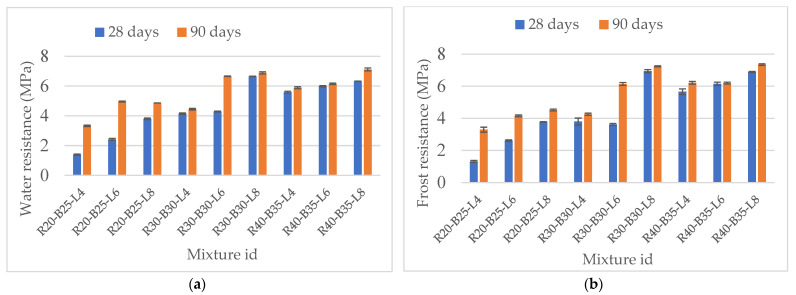
Results: (**a**) water resistance and (**b**) frost resistance.

The study of water and frost resistance of the developed compositions was studied in accordance with the requirements of the technical conditions of the state standard of the Republic of Kazakhstan ST RK 973-94 “Stone materials and soils treated with inorganic binders for road and airfield construction” for strengthening soils with slags, after hydration and hardening after 28 and 90 days. According to the building regulations of Kazakhstan, water-resistance and frost resistance of samples meets the requirements except for sample R20-B25-L4. The remaining samples meet the requirements of the first strength class (4–6 MPa) of water-saturated samples and the value of the frost resistance coefficient. The water-resistance coefficient of materials after 90 days of hydration was observed to be 1.13. The coefficient of frost resistance after 25 cycles of freezing-thawing was observed up to 1.14. An increase in the parameters of hardening of materials for water resistance from 28 to 90 days is proof of strengthening the structure of the samples. The strength values of the samples after 24 h of full water saturation fluctuate within 1.39–6.32 MPa at 28 days and 3.33–7.12 MPa at 90 days. The strengths for frost resistance values of the samples were 1.3–6.89 MPa in 28 days and 3.3–7.35 MPa after 90 days. The water resistance of materials increases simultaneously with an increase in their strength and changes in the coefficient of linear expansion of the samples. The frost resistance coefficient after 25 freeze-thaw cycles is in the range of 0.70–0.89, which also significantly exceeds the requirements. During each of the 25 expansions of the samples due to the crystallization of water into ice particles, the volume of materials expands and microcracks and microvoids form throughout the volume of the samples. When melting ice crystals are immersed in water at + 25 °C for 8 h, they leave pores that fill with water and hydrate new amounts of alkaline elements, causing a second wave of synthesis of sol-gel neoplasms that strengthen the samples. The composition R40-B35-L8 exhibited the highest results of water resistance and frost resistance. With the introduction of highly alkaline RM sludge into the soil, the strength under uniaxial compression increases as well as the samples’ water and frost resistance. The data in Figure 8 shows that with the increasing strength of materials, their water and frost resistance also increase. Mymrin (2015) [63] investigated and explained the reason for the coefficient of frost resistance value exceeding one: The phenomenon of micro-desquamation of slag particles was caused by temperature shocks, as well as increased surface hydration, alkaline corrosion, and the synthesis of new formations that strengthen the samples over 25 freezing and thawing cycles.

The standard deviations of the water resistance and frost resistance increase with axial resistance. The standard deviations of the test samples were low and varied between 0.01 and 0.09. A similar increase in the mechanical properties of concrete is observed when testing the frost resistance of materials. The standard deviations of the test samples were low and varied between 0.01 and 0.21. This significantly affected the improvement of the strength properties of concrete [64].

#### 3.2.4. Changes in the Carbonate Content of Materials during Hydration of Samples

**Figure 9:**
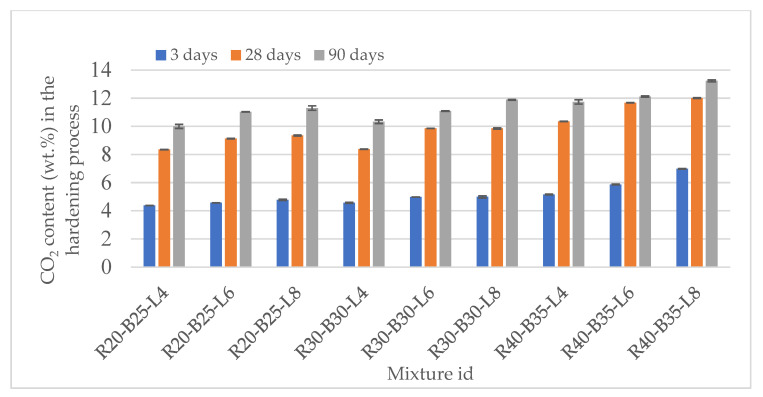
Change in the carbonate content of materials.

Figure 9 illustrates the carbonatization of the studied compounds, which begins immediately after the mixtures are hydrated. The amount of carbon dioxide absorption from the atmosphere is directly proportional to the amount of calcium oxide injected and the time after hydration begins. The change in the carbonate content of samples was determined by the weight loss method using an interaction with hydrochloric acid (HCl) in a calcimeter. According to Figure 9, it can be seen that the synthesis of crystalline or amorphous calcite cannot provide such increases in the strength of samples. The carbonate content values of the samples ranged from 4.38 to 6.98% at 3 days, 8.35 to 12.01 at 28 days and 10.8 to 13.23% at 90 days. The standard deviations of the carbonate content were low and varied between 0 and 0.16. The maximum values of standard deviations are observed in the samples R20-B25-L8, R30-B30-L4, and R40-B35-L4. The process of adsorption of CO_2_ in the air gradually reduces its intensity between 28 and 90 days. The formation of Ca (OH)_2_ mineral portlandite reduces the porosity of materials. A decrease in the porosity of the samples and the formation of a shell around lime particles as the chemically most active component of the initial mixture leads to a decrease in the intake of air and the CO_2_ contained in it. The growth of carbonates in the samples contributes to an increase in strength. The further strength gain may occur due to the compaction of previously formed sol-gel structures. It is possible to assume the synthesis of other new formations, such as amorphous compounds of the C-S-H group of a changing ratio of elements. The amorphous gel that forms between the particles in the beginning materials, in accordance with the principle of amorphous hardening, strengthens the sample’s bulk.

By using waste as a valuable raw material and displacing relatively expensive and increasingly scarce pure natural materials, it will be possible to put an end to industrial dumps that pollute the environment in Kazakhstan as soon as possible if the findings of these studies are applied at the industrial level. Developed construction materials may have a good application in the market for construction materials because of their lower costs in current processes and may play an important role in eliminating the storage of production waste in Kazakhstan.

## 4. Conclusions

The combination of RM, BFS, and LPW showed positive results in the strength and durability of NL in both dry and wet environments. The mechanical properties, hydration characteristics, and environmental friendliness performance of the production waste based road base material were studied. The study of their properties begun in the early stages (up to 90 days inclusive) of hardening of samples. Based on the experimental results, the following conclusions can be made:

1. The possibility of developing materials for road construction based on various industrial wastes (RM, BFS, and LPW) and NL was experimentally confirmed. In order to achieve maximum mechanical properties in the developed composites, LPW additives were used as a highly alkaline activator of the chemical interaction between BFS and NL.

2. The strength, mechanical properties, and durability improved with the increased ratios in the mixture. R40-B35-L8 exhibited the highest values of physical properties with maximum amounts of slag BFS (35%), RM (40%), and LPW (8%). The compressive strength of the samples reached up to 7.38 MPa at 90 days; judging by the dynamics, it will continue to grow, but at a slower pace.

3. Industrial waste materials such as RM, BFS, and LPW can be attractive due to the simplicity of the technological process for strengthening NL. The linear expansion coefficient slowly increased from 0.63 to 1.65% at 90 days. The coefficient of linear expansion of samples begun to decrease due to gel compaction during syneresis.

4. An increase in the parameters of hardening of materials for water resistance from 28 to 90 days is proof of strengthening the structure of the samples. With the introduction of highly alkaline RM sludge into the soil, the strength under uniaxial compression increased as well as the samples’ water and frost resistance. The materials showed high water and frost resistance, met the requirements of top building materials, and had significantly lower linear expansion values at all stages of their strengthening. The samples’ strengths for water resistance values were 1.39–6.32 MPa at 28 days and 3.33–7.12 MPa at 90 days. The strengths for frost resistance values of the samples were 1.3–6.89 MPa at 28 days and 3.3–7.35 MPa at 90 days.

5. The calculated carbonate content values of the samples were 4.38–6.98% at 3 days, 8.35–12.01 at 28 days, and 10.8–13.23% at 90 days. The process of adsorption of CO_2_ in the air gradually reduced its intensity between 28 and 90 days. The growth of carbonates in the samples contributed to an increase in the strength of the samples.

6. In addition to a significant increase in the strength of materials, the environment protection goal was achieved—the disposal of hazardous industrial waste—from processing bauxite RM with a high content of hazardous chemical compounds. Waste utilization positively impacts the environment since it reduces waste disposal, extends the life of industrial landfills, and reduces environmental harm caused by natural material extraction. Consequently, the developed materials significantly contribute to environmental conservation. The low cost of industrial waste used in research and the possibility of using it in large quantities makes it economically feasible compared to traditional natural raw materials. These materials allow a reduction in the irreversible destruction of natural binders in quarries for the extraction of natural building materials.

## Figures and Tables

**Table 1:** The mixing combination of the material.

Samples	Mix Id	Compositions, wt.%				
		Natural Loam	Red Mud	Blast Furnace Slag	Lime Production Waste	Moisture Content
1	R20-B25-L4	51	20	25	4	10–12
2	R20-B25-L6	49	20	25	6	10–12
3	R20-B25-L8	47	20	25	8	10–12
4	R30-B30-L4	36	30	30	4	10–12
5	R30-B30-L6	34	30	30	6	10–12
6	R30-B30-L8	32	30	30	8	10–12
7	R40-B35-L4	21	40	35	4	10–12
8	R40-B35-L6	19	40	35	6	10–12
9	R40-B35-L8	17	40	35	8	10–12

**Table 2:** Main elements of raw materials.

Raw Materials	Elements, wt.% (Average Value)										
	O	Mg	Na	Ca	K	Si	S	Al	Ti	Mn	Fe
RM	40.53	0.18	1.84	28.58	-	7.35	0.24	2.12	1.52	0.17	17.47
BFS	40.57	5.01	0.30	26.51	0.68	14.64	1.05	5.22	0.38	0.52	5.12
LPW	42.25	0.41	-	57.07	-	0.15	-	0.11	-	-	-
NL	47.28	1.55	0.45	7.78	2.81	25.26	-	8.37	0.37	0.19	5.92

**Table 3:** Chemical composition of the raw materials.

Raw Materials	Oxides, wt.%												
	Al_2_O_3_	Fe_2_O_3_	CaO	K_2_O	MgO	MnO	Na_2_O	SiO_2_	SO_3_	TiO_2_	C.L. *	CO_2_ **	Σ
RM	4.57	25.39	45.15	-	0.33	0.26	2.81	17.59	0.67	2.90	0.33	0.86	100.00
BFS	10.02	6.70	37.75	0.84	8.42	0.68	0.41	31.86	2.67	0.64	0.65	5.65	100.00
LPW	0.26	-	98.52	-	0.84	-	-	0.41	-	-	0.16	7.77	100.00
NL	16.41	7.94	11.43	3.57	2.67	0.26	-	56.45	-	0.65	0.62	4.86	100.00

* C.L.—calcination loss, ** CO_2_—calcimeter method.

**Table 4:** Solubility and leaching of toxic metals in RM and BFS.

Metals	RM		BFS		SanPiN * 2.1.7–2010
	Leaching, (mg/L)	Solubility, (mg/L)	Leaching, (mg/L)	Solubility, (mg/L)	
As	0.39	<0.001	0.20	<0.001	10
Pb	<0.11	<0.1	<0.1	<0.1	250
Hg	<0.05	<0.05	<0.05	<0.05	15
Cr	<0.1	<0.01	<0.01	<0.001	1000
Ba	<0.05	<0.05	<0.05	<0.005	-
Cd	<0.01	<0.002	<0.01	<0.002	15
Al	0.50	0.85	<0.10	<0.10	-
Cu	<0.05	<0.05	<0.05	<0.05	750
Fe	0.56	0.74	0.30	0.20	-
Ni	<0.05	<0.05	<0.05	<0.05	200
Zn	<0.10	<0.10	<0.10	<0.10	1750

* SanPiN 2.1.7.–2010. Sanitary and epidemiological rules and regulations.

**Table 5:** pH values of raw materials.

PH Value of Raw Materials			
NL	BFS	RM	LPW
7.3	8.5	9.6	12.10

**Table 6:** Granulometric fractions of the materials.

Raw Materials	Granulometric Fractions							
	Mkm	<1	1–5	6–10	11–50	51–100	101–250	>250
RM	Weight %	2	46	5	20	11	10	6
FS		10	9	10	42	11	8	10

## Data Availability

The data presented in this study are available on request from the corresponding author.

## Abbreviations

RM	Red mud
BFS	Blast furnace slag
SS	Steel slag
NL	Natural loam
LPW	Lime production waste
SEM	Scanning electron microscope
XRD	X-ray Diffraction
XRF	X-ray fluorescence
EDS	Energy dispersive spectroscopy
AAS	Atomic absorption spectroscopy
